# Gel immersion EMR for hemorrhagic gastric hyperplastic polyp

**DOI:** 10.1016/j.vgie.2024.03.012

**Published:** 2024-03-26

**Authors:** Naoki Akizue, Kenichiro Okimoto, Tomoaki Matsumura, Keisuke Matsusaka, Jun Kato, Naoya Kato

**Affiliations:** 1 Department of Gastroenterology, Graduate School of Medicine, Chiba University, Chiba, Japan; 2 Department of Diagnostic Pathology, Graduate School of Medicine, Chiba University, Chiba, Japan; 3 Department of Gastroenterology, Graduate School of Medicine, Chiba University, Chiba, Japan; 4 Endoscopy Center, Chiba University Hospital, Chiba, Japan; 5 Department of Gastroenterology, Graduate School of Medicine, Chiba University, Chiba, Japan

## Introduction

Gastric hyperplastic polyp is a frequent cause of bleeding and amenable to endoscopic resection (ER). In cases of positive *Heliobacter pylori* infection, eradication may result in reduction or disappearance of polyps,^1^ but ER is sometimes required. Although there have been reports of gel-immersion EMR being performed for gastric lesions,^2^^,^^3^ there are no reports about gel-immersion EMR for hemorrhagic hyperplastic polyps. Viscoclear (Otsuka Pharmaceutical Factory, Tokushima, Japan) is a new gel without electrolytes that consists of xanthan gum, locust bean gum, concentrated glycerin, and purified water. This was approved only in Japan in 2020, not approved by the U.S. Food and Drug Administration. The gel used for gel-immersion EMR is highly viscous and easily fills the peri-lesion area, creating a space for endoscopic visualization and treatment. Furthermore, bleeding from the resection site is less likely to spread due to the effect of a loss tangent in Viscoclear,^4^ making it easier to maintain a clear field of view afterward. Here, we present a case in which gel-immersion EMR with electrocautery was useful for a hemorrhagic hyperplastic polyp with bleeding right after resection (Video 1, available online at www www.videogie.org).

## Case Presentation

**Figure 1:**
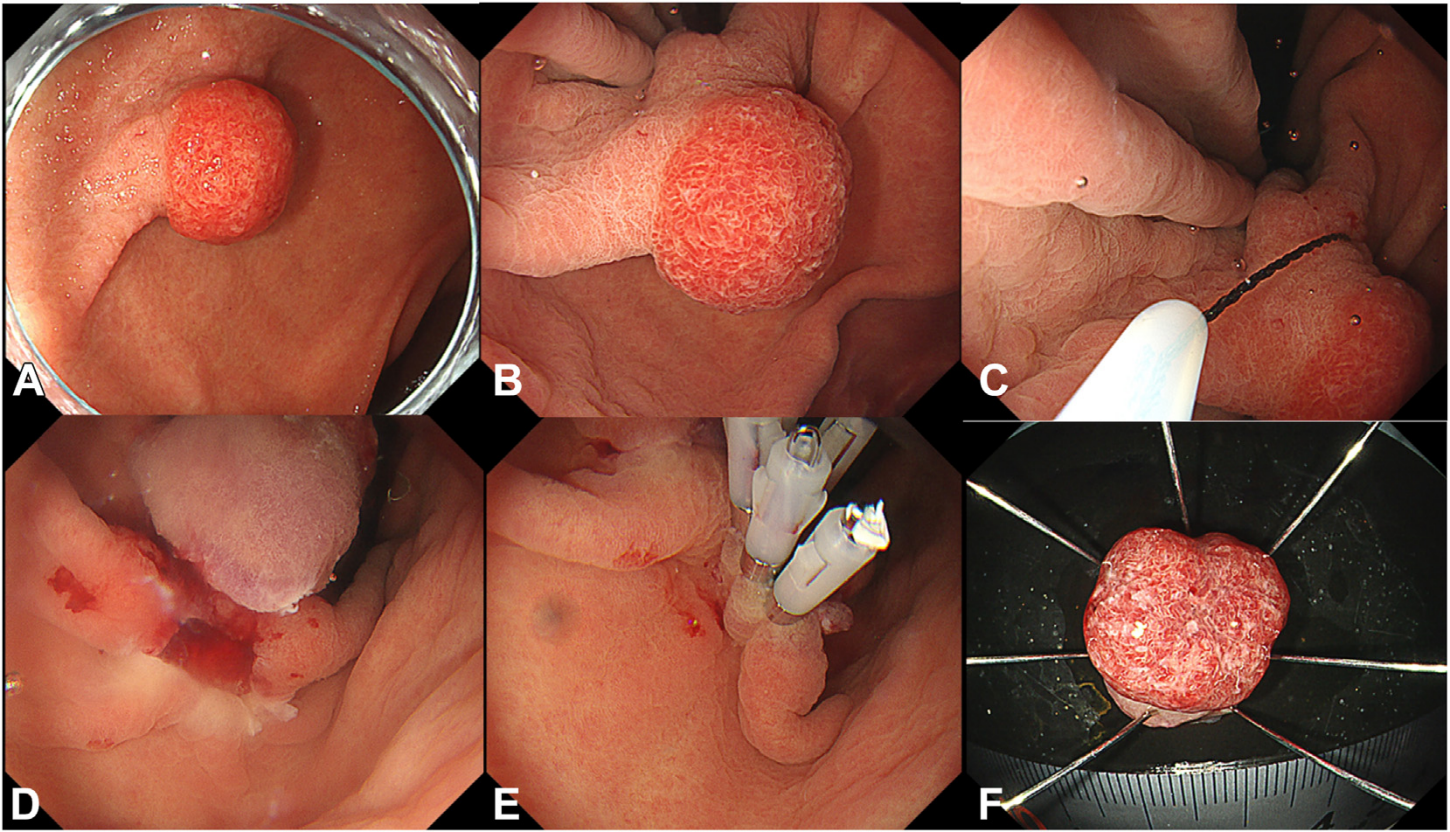
Images of gel-immersion EMR for the gastric polyp. **A,** Endoscopic image of the gastric polyp at the lesser curvature of the antrum. **B,** Endoscopic image of the gastric polyp observed under gel immersion. **C,** Under-gel condition made it easy to recognize the margin and snare the polyp. **D,** Bleeding from the post-resected defect did not spread, owing to a clear field of view. **E,** Clip closure was successfully performed. **F,** The histopathological diagnosis was foveolar hyperplastic polyp.

A 79-year-old male patient with anemia underwent EGD, revealing a 10-mm polyp with bleeding located at the lesser curvature of the antrum (Fig. 1A). The biopsy results showed a hyperplastic polyp. As water immersion was difficult due to the location, gel-immersion EMR instead of underwater EMR (UEMR) using Viscoclear was selected. Viscoclear can be used by simply sucking the product out of the package and injecting it directly into the endoscope’s working channel. A total of 150 mL of gel was sufficient to immerse the polyp with good visualization (Fig. 1B). Owing to the effect of the gel, the polyp floated up in the lumen, making it easy to recognize the margin and snare the polyp (Fig. 1C). Successful en bloc resection was achieved. Despite minor bleeding from the post-ER defect, the blood under the gel did not spread, maintaining a clear visual field (Fig. 1D). As a result, it was easy to close the ulcer with clips (Fig. 1E). The histopathological diagnosis was a foveolar hyperplastic polyp (Fig. 1F). The follow-up endoscopy has not been conducted, as 1 year has not yet passed.

## Discussion

Conventional EMR (CEMR) has been used widely for gastric polyps, but CEMR is costly in that it requires submucosal injection. CEMR requires an injection needle that costs about $50, whereas gel-immersion EMR requires Viscoclear, which costs approximately $15. The need of clips after removing polyps under 20 mm in both CEMR and gel-immersion EMR remains controversial. Although avoiding clip (approximately $5) use can reduces costs, even when using a few clips, gel-immersion EMR is deemed more cost-effective than CEMR. UEMR can be an option^5^^,^^6^ but may be sometimes difficult to achieve adequate underwater conditions due to the localization, such as the lesser curvature of the body to the antrum. We think that these locations are more favorable for gel-immersion EMR.

In gel-immersion EMR, similar to UEMR, the lumen is degassed and flooded. This process relaxes the mucosa and causes the tumor to float up inside the lumen, enabling easy strangulation of the lesion without increasing its size.^7^ Additionally, even in areas where water does not easily accumulate, gels can be stored easily, providing a good field of view for effective treatment.^8^ Moreover, gel-immersion EMR is simple method and does not use local injection. Therefore, it is relatively easy for a trainee. Another advantage is that there is no poor visibility in some cases with bleeding after resection, and hemostasis and clipping can be performed easily.

## Conclusions

Gel-immersion EMR was useful for hemorrhagic hyperplastic polyps and provided a clear field of view even if there was bleeding after resection.

## Disclosure

The authors disclosed no financial relationships relevant to this publication.

## Supplementary data

Video 1 Gel-immersion EMR for hemorrhagic gastric hyperplastic polyp.

## References

[1] Nam S.Y., Park B.J., Ryu K.H. (2018). Effect of *Helicobacter pylori* eradication on the regression of gastric polyps in National Cancer Screening Program. Korean J Intern Med.

[2] Kimura H., Oi M., Morita Y. (2022). Gel immersion endoscopic mucosal resection for a gastric neoplasm with a background of fundic gland polyposis. Endoscopy.

[3] Miura K., Sudo G., Saito M. (2022). Gel immersion endoscopic mucosal resection for early gastric cancer near the pyloric ring. Endoscopy.

[4] Yano T., Ohata A., Hiraki Y. (2021). Development of a gel dedicated to gel immersion endoscopy. Endosc Int Open.

[5] Kono Y., Sakae H., Okada H. (2018). Underwater endoscopic mucosal resection for gastric polyp. Dig Endosc.

[6] Yamamoto S., Takeuchi Y., Uedo N. (2022). Underwater endoscopic mucosal resection for gastric neoplasms. Endosc Int Open.

[7] Uedo N., Nemeth A., Johansson G.W. (2015). Underwater endoscopic mucosal resection of large colorectal lesions. Endoscopy.

[8] Amino H., Yamashina T., Marusawa H. (2021). Under-gel endoscopic mucosal resection without injection: a novel endoscopic treatment method for superficial nonampullary duodenal epithelial tumors. JMA J.

